# Second line anti-retroviral therapy failure in a pediatric cohort of an Ethiopian tertiary hospital: a retrospective observational study

**DOI:** 10.1038/s41598-020-65714-6

**Published:** 2020-05-26

**Authors:** Tinsae Alemayehu, Workeabeba Abebe

**Affiliations:** 1 American medical center – Specialty clinic for infectious diseases and travel medicine, Addis Ababa, Ethiopia; 2 Department of Pediatrics and child health, College of health sciences, Addis Ababa University, Addis Ababa, Ethiopia

**Keywords:** HIV infections, Paediatric research

## Abstract

Sub-Saharan Africa carries the largest burden of pediatric HIV infection. The success of second line anti-retroviral treatment and related factors among African children is not well-defined. Objectives: We aimed to identify the rate and determinants of second line anti-retroviral treatment failure among children and adolescents on follow-up at an Ethiopian tertiary teaching hospital. A retrospective observational cohort study was conducted at Tikur Anbessa Specialized Hospital, Addis Ababa. Structured forms were used to collect socio-demographic, clinical and diagnostic data. Descriptive statistics and bivariate analysis were used to describe the magnitude of the problem and its associations. A total of 76 children and adolescents taking second line anti-retroviral treatment were analyzed (mean age: 16.1 years). Failure of therapy was seen in 14/76 while four were eligible for a switch to third line anti-retrovirals. Mean duration on second line treatment till virologic failure was diagnosed was 17.6 months while mean viral load upon requiring a third line regimen was 82,131.3 copies/ml. Second line antiretroviral treatment virologic failure was significantly associated with the age of the child or adolescent. A high rate of virologic failure exists among the study population. Findings underline need for provision of third line anti-retroviral drugs in Ethiopia. Challenges for delivering a standard care were irregular viral load testing and delayed initiation of second line treatment after failure of first line regimens.

## Introduction

Anti-retroviral treatment (ART) free of charge has been provided in Ethiopia since 2005. At present, nine anti-retroviral drugs (ARVs) are prescribed as first and second line drugs within the country. There is a wealth of experience and data concerning the success of first line anti-retroviral treatment in Ethiopia. Determinants for its implementation have also been well studied^1–5^.

Thorough investigation of second line anti-retroviral treatment failure has enabled developing countries to improve access to third line ARV regimens while avoiding early, unnecessary switching too^6^. A systematic review on second-line antiretroviral treatment failure in all age groups in sub-Saharan African showed a pooled rate of 15 per 100 person years – with children, residents of Southern Africa and those on therapy for 12–18 months being at particular risk^7^. Another report from a rural South African clinic found that virologic failure for second line regimens stood at 12.9 per 100 person years among adults^8^.

Among the adult population living with HIV in the region, advanced immunosuppression, depression, non-adherence and use of herbal medicine are associated with failure of viral suppression using second line regimens^7,9^. Studies among adult subjects in Uganda confirmed that delayed switch to second line therapy impaired its efficacy^10^. Similar studies also suggest that the addition of a protease inhibitor (PI) as a component of second line therapy on to nucleoside reverse-transcriptase inhibitor (NRTI) -based regimen conferred better disease control^11^.

There is a paucity of similar data from children and adolescents in Africa. Our study focused on determining the rate and predictors for treatment failure for second line ART among children and adolescents living with HIV and on follow-up at Ethiopia’s largest tertiary care referral center – Tikur Anbessa Specialized Hospital located in the capital city of Addis Ababa.

## Method

### Study setting

The study was conducted at the Pediatric Infectious Diseases’ Clinic (PIDC) located at Tikur Anbessa Specialized Hospital (TASH), Addis Ababa. TASH is a government-ran tertiary teaching hospital which gives treatment to more than 30,000 patients annually. Its PIDC evaluates an average of 350 children per month with communicable diseases. It also gives comprehensive HIV treatment and care to 512 HIV infected children and adolescents, 100 of whom are on second line ART.

### Study design and period

The research is a retrospective observational cohort study of patients suspected of having second line anti-retroviral treatment (ART) failure and had been followed for at least six months. Data was collected from January to August 2018 on children and adolescents who had been on follow-up at the clinic from January 2006 – August 2018.

### Study population and sampling

The study population were children and adolescents on a second line ART regimen after a switch based on criteria recommended by the Ethiopian national ART treatment and care guidelines^12^. Accordingly, all under care and aged 18 years or less based on the World Health Organization (WHO) definitions of a child and an adolescent with HIV infection diagnosed by either by serological method or by Deoxyribonucleic acid Polymerase chain reaction (DNA PCR) test and who had been on second line ART therapy for at least six months were included. We also restricted inclusion to those who had at least one viral load determination during their follow-up. All others who were not taking second line ARVs, had been taking them for less than six months or had no form of follow-up viral load testing were excluded.

### Data collection

Data collection took place in the period between January and August 2018. Following de-identification of study population, patient charts, pre-ART registers and ART registers were utilized to extract sociodemographic, clinical and diagnostic data. Those were then documented on structured data abstraction forms.

Independent variables were age, gender, nutritional status (Change in weight), type of first line and second line ARV drugs with baseline clinical and laboratory findings, stage of illness, viral load and CD4 measurements, duration on first and second line ART, and adherence. The dependent variable was failure of second line ART based on virologic criteria.

Second-line ART was defined as the regimen used for treatment among people living with HIV who failed their first-line regimen based on the Ethiopian national ART treatment and care guidelines^12^. Eligibility for third line ART was defined as HIV viral load of greater than 1000 copies/mL after at least six months on second line ART, confirmed on two consecutive viral load (VL) measurements three months apart, with adherence support while an undetectable VL was taken as a VL less than 50 copies/ml. Failure of second line ART was taken as at least one elevation in viral load above 1000 copies/mL among those who had at least one determination of viral load during follow-up for second line ART and failed to show up for follow-up sessions to confirm failure of virologic suppression). Adherence (based on national protocols) was assessed using parent or caretaker or child or adolescent’s report as well (more than 95% pills taken), fair (85–95% pills taken) and poor (less than 85% pills taken)^12^.

### Statistical analysis

All recorded data were analyzed by the SPSS version 23.0 software. Descriptive statistics for baseline characteristics and bivariate analysis were used. Variables which were found to be associated with increased treatment failure in bivariate analysis were included in multivariate analysis for adjustment of potential confounders. 95% confidence intervals were determined and a P-value of less than 0.05 was considered statistically significant for association with treatment failure.

### Ethical considerations

All research protocols were approved by the Research and Publication Committee of the Department of Pediatrics & Child Health, College of Health sciences, Addis Ababa University. The committee had confirmed that consent from the participants or their parents/guardians was not necessary as data was to be collected from charts without the inclusion of patient identifiers.

## Results

From a pool of 512 children and adolescents under follow up in the clinic, 100 who were under second line HIV treatment and care were screened. Among these, 76 had at least one determination of viral load during follow-up for second line ART (Table 1). Their mean age was 16.1 years while their residential areas spread across all ten sub-cities of Addis Ababa and surrounding towns. A majority on follow-up had lost one or both parents (51/76).

**Table 1:** Demographic/clinical characteristics of children/adolescents taking 2^nd^ line anti-retroviral drugs at the Pediatric infectious diseases’ clinic, Tikur Anbessa specialized hospital, Addis Ababa, Ethiopia.

Characteristic		Study population (n = 76)	
		Treatment failure (n = 14)	No treatment failure (n = 62)
Gender	Female (n = 29)	6	23
	Male (n = 47)	8	39
Age group	5–10 years (n = 4)	3	1
	10–15 years (n = 28)	4	24
	15–18 years (n = 44)	7	37
Parents	Both alive (n = 23)	4	19
	One alive (n = 27)	4	23
	Both dead (n = 24)	6	18
	Undocumented (n = 2)	0	2
Primary caregiver	Parent/s (n = 46)	7	39
	Sibling/s (n = 3)	2	1
	Uncle/aunt (n = 7)	2	5
	Grandparents (n = 2)	1	1
	Orphanage (n = 6)	1	5
	Other (n = 12)	1	11
Taking Trimethoprim-Sulfamethoxazole prophylaxis	Yes (n = 26)	7	19
	No (n = 50)	7	43
Taking or has completed Isoniazid prophylaxis therapy in past 3 years	Yes (n = 62)	13	49
	No (n = 14)	1	13
Viral set-point at start of 2^nd^ line ART (copies/ml)	1000–50000 (n = 27)	5	22
	More than 50000 (n = 17)	4	13
	Not tested upon initiation (n = 32)	5	27
Disclosure status	Undisclosed (n = 1)	0	1
	Disclosure level 2 (n = 6)	1	5
	Disclosure level 3 (n = 68)	13	55
	Undocumented (n = 1)	0	1
Adherence	Well (n = 71)	13	58
	Fair (n = 1)	0	1
	Poor (n = 4)	1	3

A majority of the study population (72/76) received first line regimens combining two Nucleoside reverse transcriptase inhibitors (NRTI) with a non-nucleoside reverse transcriptase inhibitor (NNRTI). None of the pediatric population on second line ARVs had been exposed to ARVs for prevention of mother to child transmission (PMTCT). The mean duration on first line HAART before diagnosing failure and switching to second line ARVs was 71.5 months. It took a mean of 144.2 days to initiate second line ARVs after confirming need for second line treatment and the mean viral load upon starting second line treatment was 140, 304.8 copies/ml.

The mean duration on second line ART was 42.5 months. Almost all of the study population (75/76) were initiated on second line regimens comprising of two NRTIs and a boosted protease inhibitor (PI: Lopinavir/ritonavir or Atazanavir/ritonavir).

The proportion of children and adolescents who failed their 2^nd^ line regimen were 14 out of 76; four of these being eligible for the initiation of a 3^rd^ line ART. The mean time till virologic failure was 17.6 months after initiating 2^nd^ line ART. The mean viral load upon requirement of a third line regimen was 82,131.3 copies/ml. Second line HAART virologic failure was significantly associated with the age of the child or adolescent (Table 2).

**Table 2:** Bi-variate analysis of determinants of failure of second line HAART among children/adolescents at the Pediatric infectious diseases’ clinic, Tikur Anbessa specialized hospital, Addis Ababa, Ethiopia.

Variables		Failure of 2^nd^ line ART		Adjusted OR (95% CI)	P value
		Yes (n = 14)	No (n = 62)		
Age group	5–10 years	3	1	0.000 (0.000–0.039)	0.011
	10–15 years	4	24		
	15–19 years	7	37		
Gender	Female	6	23		0.689
	Male	8	39		
Parents	Both alive	4	19	0.684 (0.580–0.789)	0.634
	One alive; the other dead	4	23		
	Both dead	6	18		
	Undocumented	0	2		
Primary caregiver	Parent/s	7	39	0.171 (0.086–0.256)	0.232
	Sibling/s	2	1		
	Aunt/Uncle	2	5		
	Grandparent/s	1	2		
	Orphanage	1	5		
	Other	1	11		
Time lapse after confirming virologic failure for 1^st^ line ART till starting 2^nd^ line ART	One month or less	14	53	0.618 (0.509–0.728)	0.512
	1–2 months	0	3		
	2–3 months	0	2		
	3 months or more	0	4		
Viral set-point at start of 2^nd^ line ART (copies/mL)	1,000–50,000	5	22	0.855 (0.776–0.934)	0.794
	More than 50,000	4	13		
	Undocumented or not tested	5	27		
Taking CPT	Yes	7	19		0.168
	No	7	43		
Took IPT within past 3 years	Yes	13	49		0.228
	No	1	13		
Disclosure status	DS2	1	5	1.000 (0.961–1.000)	0.922
	DS3	13	55		
	Undisclosed	0	1		
	Undocumented	0	1		
Adherence	Good	13	58	1.000 (0.961–1.000)	0.843
	Fair	0	1		
	Poor	1	3		

Key: ART – Anti-retroviral treatment, CPT – Co-trimoxazole prophylactic treatment, IPT – Isoniazid prophylactic treatment, DS – Disclosure status, VL – Viral load.

None were started on third line regimens as they were unavailable. Resistance testing for ARVs is not available in the hospital. Viral load testing was performed during only 37.8% (108/286) of clinic visits scheduled every six months while on second line ARVs.

## Discussion

Our study showed 100 children and adolescents were switched to second line ART among our cohort of 512 (19.5%). This was comparable to Okechukwu *et al*.'s observation in Abuja, Nigeria with 15.9% of their pediatric population living with HIV having been switched to a second line regimen^13^. But the latter study was performed in 2015 and as such, the proportion for whom a 1^st^ line ART was switched could be higher at present. The mean age of children/adolescents on second line therapy was 16 years which is to be expected as it co-exists with prolonged life expectancy while on HIV treatment and care. There was a marked delay in diagnosing failure of a first line regimen (71.5 months). This fares worse than seen among studies in South Africa (22.2 months), Thailand (23 months) and Nigeria (31.3 months)^13–15^. A similar delay was seen in initiating a second line treatment once failure of first line regimens was diagnosed (144.2 days) which mirrors Davies *et al*.'s observation of a 171 day delay in a multi-center study done among South African hospitals^16^. Though widely available at present, the initial low stocks of second line regimens and uninformed practices leading to tolerance of failed regimens may have been determining factors for the delay. Postponement of switch to second line therapy is related with poor outcomes among populations living with HIV. In South African patients, a 1.47 times higher risk of death was shown when switch to 2^nd^ line anti-retroviral drugs was delayed by 6–12 months (as compared to 0–1.5 months) after diagnosis of first line treatment failure. There was also a 2.13 times higher rate of second-line treatment failure when switch was delayed by 3–6 months (as compared to 0–1.5 months)^17^. The mean viral load upon initiation of 2^nd^ line ART was 140,304.8/mL. A study performed among Nigerian children showed the mean VL upon switch to second line ARVs was 180,480.3 copies/mL^12^. A viral set-point of more than 100,000/mL at start of second-line treatment has been confirmed to correlate with a 2.1 times higher risk of mortality^18^. Overall, 14 of the 76 those under care with second line ART had failed their treatment; four of those meeting criteria set by the national guidelines as well as WHO for eligibility for third line ARVs. This figure was comparable to that reported from a Ugandan pediatric cohort, where 20% of analyzed children had virologic failure for second line ARVs^19^ but significantly less than that reported from a multi-center study in Thailand (49% failure while on PI-based second line therapy)^14^. Only 37% visits made every 6 months apart while under second line ART were accompanied by viral load measurements. This may be due to absence of children upon scheduled visits and inconsistencies in facility labs and may have underestimated the actual rate of failure for second-line therapy. The mean viral load upon those requiring a third line regimen was 82,131.3 copies/ml. The corresponding counts from Okechukwu *et al*. in their study in Abuja teaching hospital, Nigerian was 237,337.5 copies/ml upon failure of second line ARVs^12^. As depicted above, a high viral set-point upon initiating third line ARVs is expected to have a deterrent effect against viral suppression and towards higher mortality.

Age of the child/adolescent was significantly associated with failure of their second line ARV regimens. Prolonged life expectancy and improvement in HIV treatment and care are expected to co-exist with failure of long-term regimens. Taking first line ART drugs for more than 2 years was found to be a significant determinant of failure of second line ART among Thai children^14^ as were age greater than 13 years and BMI for age Z score less than −2 SD at initiation of second line ARVs.

## Conclusion

In conclusion, our study shows a high rate of virologic failure among children and adolescents on second line ARVs (one in five) and need for third line ARVs in our hospital. Failed first line regimens were tolerated for long leading to high viral set-points at start of second line therapy. Early detection of first line treatment failure and switch of therapy prolongs effective lifespan of the second line ARVs. A similar high viremia was noted for those children and adolescents who had failed their second line regimen. The viral load monitoring was sub-standard and is likely to have underestimated rates of second line treatment failure. A short time lapse after starting second line ARVs till diagnosis of failure was noted in instances of virologic failure. Better adherence counseling and making third line an-retroviral drugs available are mandatory to continue on the gains achieved in life expectancy in this population.

### Ethical considerations

All research protocols were approved by the Research and Publication Committee of the Department of Pediatrics & Child Health, College of Health sciences, Addis Ababa University. The committee had confirmed that consent from the participants or their parents/guardians was not necessary as data was to be collected from charts without the inclusion of patient identifiers.

### Consent for publication

All research protocols were approved by the Research and Publication Committee of the Department of Pediatrics & Child Health, College of Health sciences, Addis Ababa University. The committee had confirmed that consent from the participants or their parents/guardians was not necessary as data was to be collected from charts without the inclusion of patient identifiers.

## Data Availability

We confirm that all data concerning the study is made available along with the submission of the manuscript.
